# Intratracheal Administration of Polystyrene Micro(nano)plastics with a Mixed Particle Size Promote Pulmonary Fibrosis in Rats by Activating TGF-β1 Signaling and Destabilizing Mitochondrial Dynamics and Mitophagy in a Dose- and Time-Dependent Manner

**DOI:** 10.3390/toxics13060487

**Published:** 2025-06-09

**Authors:** Shuang Xia, Chunli Yuan, Wei Long, Zongcheng Wu, Xiuqin Li, Nan Wang, Mumu Gao, Zhe Li, Peilun Li, Peng Liu, Xiaoxi Qu, Lina Sun

**Affiliations:** 1 Key Lab of Eco-Restoration of Regional Contaminated Environment, Ministry of Education, Shenyang University, No. 21 Wanghua South Street, Dadong District, Shenyang 110044, China; 18940115600@163.com (S.X.); chunlihappygirl@126.com (C.Y.); liasdf042@126.com (X.L.); 2 Shenyang Medical College, Shenyang University, No. 146 Huanghe North Street, Yuhong District, Shenyang 110034, China; wei200430@126.com (W.L.); 13940472359@163.com (P.L.); 3 Shenyang SYRICI Testing Co., Ltd., No. 600 Shenliao Road, Tiexi District, Shenyang 110141, China; wuzongcheng77@163.com; 4 Shenyang Ecological and Environmental Monitoring Center of Liaoning Province, No. 98 Quanyun 3rd Road, Hunnan District, Shenyang 110013, China; 18540256071@163.com (N.W.); mm0208@163.com (M.G.); cuihuahuaxue@163.com (Z.L.); 5 School of Pharmacy, Shenyang Pharmaceutical University, 103 Wenhua Road, Shenhe District, Shenyang 110016, China; pengliu@syphu.edu.cn; 6 Joint International Research Laboratory of Intelligent Drug Delivery Systems, Ministry of Education, No. 103 Wenhua Road, Shenhe District, Shenyang 110016, China; 7 Shenyang Women’s and Children’s Hospital, Delivery Room, 87 Danan Street, Shenhe District, Shenyang 110011, China; 13478352580@163.com

**Keywords:** microplastics, pulmonary fibrosis, inflammation, mitochondrial dynamics, mitophagy, benchmark dose

## Abstract

Background: Microplastics (MPs) can be inhaled by people. However, the relationships between long-term exposure to inhaled MPs, pulmonary fibrosis, and mitochondrial dysfunction are not completely clear. Methods: SD rats were exposed to a 0.0125, 0.125, 0.31, or 1.25 mg/day dosage of mixed polystyrene MPs (PS-MPs), with the particle sizes ranging from 500 nm to 4 µm, via intratracheal administration, for 7 to 35 consecutive days. Results: PS-MPs with particle sizes ranging from 1 µm to 4 µm were deposited in the lungs. The contents of NFκB-mediated proinflammatory cytokines were increased in the lungs of the rats after 7 days of PS-MP exposure. After exposure to PS-MPs, the degree of collagen deposition and the expression of TGF-β1/Smad increased significantly, and the levels of phosphorylated Akt (p-Akt) and nuclear β-catenin decreased significantly. The number of healthy mitochondria decreased, the expression of mitochondrial fission and fusion proteins increased, and the level of PINK1/Parkin-mediated mitophagy decreased in the lungs of the rats after 7 days of PS-MP exposure. A benchmark dose (BMD) of 0.151 mg/day and a benchmark dose lower confidence limit (BMDL) of 0.031 mg/day were identified on the basis of the subchronic effects of the intratracheal administration of the PS-MPs. Conclusions: Our study provides an in-depth understanding of the potential impacts of MP pollution on respiratory diseases.

## 1. Introduction

Plastics are widely used in daily life because of their light weight, chemical corrosion resistance, and low manufacturing costs. However, plastics are also resistant to biodegradation in the environment. Plastics are broken down into small particles by light, heat, radiation, and mechanical forces. Plastics with a particle size less than 5 mm are defined as microplastics (MPs) [1], which have a stronger ability to absorb pollutants because of their large specific surface area [2]. High-density polyethylene, low-density polyethylene, polyethylene terephthalate, polypropylene, polystyrene (PS), polyvinyl chloride, and polyamide are commonly found in water, soil, and the atmosphere and can be absorbed by the human body, mainly through drinking water or diet, the respiratory system, and direct contact with the skin [3,4,5]. MPs can be easily transferred into the air. In contrast to MPs in other media, airborne MPs can directly and continuously enter the human body and induce severe lung damage.

Within human lung tissue, MP particles are smaller than 5.5 µm [6], and MP fibers have a mean length of 223.10 ± 436.16 μm and a mean width range of 22.21 ± 20.32 μm [7]. These MPs can accumulate in the lungs for 180 days and cause continuous lung damage and even pulmonary fibrosis [8]. Li X et al. (2022) reported that the inhalation of polystyrene microplastics (PS-MPs), with a diameter of 5 µm, at a dose of 6.25 mg/kg, three times per week, can trigger oxidative stress in C57BL/6 mice [9]. Fan Z et al. (2022) reported that the intratracheal instillation of 100 nm of PS-MPs increased the levels of interleukin-6 (IL-6), tumor necrosis factor α (TNF-α), interleukin-8 (IL-8), and interleukin-1β (IL-1β) in the bronchoalveolar lavage fluid of SD rats [10]. Dong CD et al. (2020) reported that PS-MPs have significant cytotoxic effects on the BEAS-2B human bronchial epithelial cell line [11]. Yang S et al. (2021) reported that, after exposure to 30 μg/cm^2^ of PS nanoplastics (NPs) with a particle size of 40 nm, 1951 genes showed expression changes in BEAS-2B cells, and the pathways associated with the genes with the most significantly altered expression were those related to the extracellular matrix (ECM)–receptor interaction and steroid biosynthesis [12]. Phosphatidylinositol-4,5-bisphosphate 3-kinase (PI3K)/protein kinase B (Akt) signaling was significantly inhibited in the skeletal muscles of mice fed with 10 mg/L of MPs in their drinking water [13]. In addition, treatment with 100 mg/L of PS-MPs in their drinking water for 6 weeks inhibited PI3K/Akt signaling in birds [14]. With respect to basic research on MPs in vivo, previous studies have focused mostly on the damage caused by MPs in terms of a single particle size. However, MPs in the atmosphere and human lungs vary in size. Therefore, the use of MPs with a single particle size may be a limitation of the existing research.

MPs are toxic to mitochondria [15,16]. PS-NPs can cause mitochondrial enlargement, crista disintegration, and a decrease in the membrane potential of Tetrahymena thermophila [17], and affect mitochondrial energy metabolism in zebrafish larvae by reducing mitochondrial NADH productivity, the embryonic mitochondrial coupling efficiency, and their respiration ability [18]. MPs can reduce the ATP content and activate mitophagy in mouse spermatocyte GC-2 cells, ultimately inducing cell death [19]. The accumulation of dysfunctional mitochondria in pulmonary fibrosis type II alveolar epithelial cells is associated with the downregulation of mitophagy [20,21].

In basic research on MP inhalation in vivo, the duration of MP exposure has typically been relatively short, ranging from a few hours to a few days. Few studies have investigated the effects of long-term exposure to MPs via inhalation or intratracheal instillation on the lungs. The effects of long-term intratracheal administration of MPs with a mixed particle size on the degree of pulmonary fibrosis and the relationships between the structure and function of the mitochondria, pulmonary mitophagy, and mitochondrial dynamics are still not completely clear.

The use of a benchmark dose (BMD) is a way to determine the probability of toxic reactions at low doses. The method used to determine the BMD involves the use of multiple statistical models to fit various continuous or dichotomous variables, form dose-response curves, and calculate the curve inflection point dose (BMD) and the benchmark dose lower confidence limit (BMDL) to evaluate the relationship between the dose and toxicity [22]. BMDs have been widely used in risk assessments for environmental contaminants [23]. To our knowledge, there are no reports on the threshold at which the intratracheal administration of PS-MPs induces lung injury in rats.

**Figure 1:**
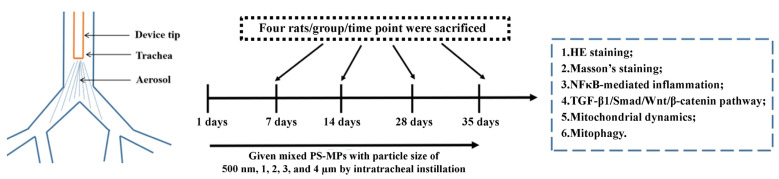
Experimental protocol.

In this study, rats were given 0.0125, 0.125, 0.31, and 1.25 mg/day doses of PS-MPs, with particle sizes of 500 nm, 1 µm, 2 µm, 3 µm, and 4 µm, mixed at a 1:1:1:1:1 weight ratio, using intratracheal aerosolization, for 7, 14, 28, and 35 consecutive days, to systematically evaluate the effects of MPs on pulmonary fibrosis and changes in the structure and function of lung mitochondria (Figure 1). The literature has reported that human inhalation doses of MPs were in the range of 6.5–8.97 µg/kg bw/day [24]. Calculated based on a weight of 70 kg, human inhalation doses of MPs were 0.455–0.628 mg/day. Therefore, the dosage range we have chosen (0.0125–1.25 mg/day) covers the environmentally relevant concentration of MPs. Furthermore, we calculated the BMD and BMDL of the intratracheal administration of PS-MPs in rats. The results of this study provide an in-depth understanding of the potential impacts of air MPs on respiratory diseases.

## 2. Materials and Methods

### 2.1. Chemicals and Reagents

Fluorescent PS-MPs, with diameters of 500 nm (red fluorescence, maximum excitation wavelength of 548 nm, 2.5 wt%), 1 µm (blue fluorescence, 383 nm, 2.5 wt%), 2 µm (blue fluorescence, 383 nm, 2.5 wt%), 3 µm (green fluorescence, 484 nm, 2.5 wt%), and 4 µm (green fluorescence, 484 nm, 2.5 wt%) were purchased from Wuxi Gerui Biotechnology Co., Ltd. (Wuxi, China). Rabbit monoclonal antibodies against p-Smad2 Ser476 (ab280888), LC3 (ab192890), P62 (ab109012), β-catenin (ab68183), Lamin B1 (ab16048), and p-Akt1 Ser473 (ab81283) and secondary antibodies were purchased from Abcam (Cambridge, UK). Rabbit polyclonal antibodies against TGF-β1 (21898-1-AP), DRP1 (12957-1-AP), Mfn1 (13798-1-AP), Mfn2 (12186-1-AP), VDAC1 (55259-1-AP), and β-actin (81115-1-RR) were purchased from ProteinTech (Wuhan, China). A rabbit polyclonal antibody against PINK1 (WL04963) was purchased from Wanleibio (Shenyang, China). A rabbit polyclonal antibody against CD86 (abs115477) was purchased from Absin (Shanghai, China). A rabbit polyclonal antibody against p-NFκB Ser536 (bs-3485R) was purchased from Bioss (Beijing, China).

### 2.2. Animals

Adult male SPF-grade Sprague–Dawley rats (7 weeks of age, 180–200 g) were procured from SiPeiFu (Beijing, China) Biotechnology Co., Ltd. The animals were housed under the following conditions: alternating light and dark conditions for 12 h each; a humidity of 45–60%, and an indoor temperature of 23 ± 2 °C. This study was approved by the institutional animal care and use committee (IACUC) of Shenyang SYRICI Testing Co., Ltd. (No. 2023CP01, approval date: 17 April 2023). All the experiments were performed in accordance with P. R. of China legislation on animal use and care and adhered to the Declaration of Helsinki and the ARRIVE guidelines.

### 2.3. Preparation of the PS-MP Mixture

MPs in the atmosphere and human lungs vary in size. Therefore, a mixture of fluorescent PS-MPs was used in this study. PS-MPs with 5 particle sizes, 500 nm, 1 µm, 2 µm, 3 µm, and 4 µm, were thoroughly mixed at a 1:1:1:1:1 weight ratio, and the amount of PS-MPs in a 50 μL mixture was 1.25 mg. The 1.25 mg PS-MP mixture was diluted 4 times with normal saline to form a mixture containing 0.31 mg of PS-MPs. The 1.25 mg PS-MP mixture was diluted 10 times with normal saline to form a mixture containing 0.125 mg of PS-MPs and then diluted another 10 times to form a mixture containing 0.0125 mg of PS-MPs.

### 2.4. Exposure Method and Treatment

Rats with similar body weights were randomly divided into the control group (no MP administration, no normal saline administration), the vehicle group (only normal saline administration), the 0.0125 exposure group (MP administration of 0.0125 mg/day/rat), the 0.125 exposure group (MP administration of 0.125 mg/day/rat), the 0.31 exposure group (MP administration of 0.31 mg/day/rat), and the 1.25 exposure group (MP administration of 1.25 mg/day/rat). The rats were subjected to intratracheal administration once a day for 5 days and were then allowed 2 days of rest every week to avoid death caused by the continuous administration of PS-MPs. Four rats from each experimental group were sacrificed with isoflurane on the 7th, 14th, 28th, and 35th day after PS-MP exposure. The body and lung weights of the rats were recorded after the rats were sacrificed. The right lungs of the rats were collected and stored at −80 °C for the PCR, WB, ELISA, and ATP measurements. Then, a 1 mm^3^ volume sample of the left lung was collected and immediately fixed with an electron microscopy fixative (Solarbio, Beijing, China) for transmission electron microscopy analysis, and the remaining lung tissue was subjected to paraffin embedding.

The following method for the intratracheal administration of PS-MPs was used. First, the rats were anesthetized with 70 mg/kg Zoletil^®^ 50 (Virbac China, Shanghai, China). The handheld liquid/dry powder aerosol lung delivery device (Beijing Huironghe Technology Co., Ltd., Beijing, China) consists of a quantitative column, an aerosol atomizing micro-nozzle (which is integrated into a stainless-steel capillary tube, the outer diameter is 0.58 mm, the inner diameter is 0.3 mm), and a high-pressure pushing device (which has a push rod). The atomization volume of the device is 50–250 μL per use. With the assistance of a laryngoscope, the slender capillary micro-nozzle is inserted into the trachea of the rats. By pushing the push rod forcefully, a 50 μL mixture of 0.0125 mg, 0.125 mg, 0.31 mg, or 1.25 mg of PS-MPs, or normal saline, was quantitatively transferred to the aerosol atomizing micro-nozzle, then atomized and transferred into the lungs.

### 2.5. Observation of PS-MPs in the Lung Tissues of the Rats

The rat lungs were fixed for more than 24 h with a 4% paraformaldehyde solution at 4 °C. The tissues were processed for paraffin embedding, according to standard procedures, and cut into 5 µm thick sections. The lung sections were deparaffinized, and the distribution of the PS-MPs in the sections was observed using a fluorescence microscope (Nikon, Y-TV55, Tokyo, Japan).

### 2.6. Hematoxylin and Eosin (HE) Staining

Five-micron-thick lung sections were deparaffinized and then stained with hematoxylin solution and eosin solution. After staining, the sections were dehydrated. Finally, the sections were observed using a microscope (Nikon, Tokyo, Japan).

### 2.7. Masson’s Trichrome Staining

Lung tissue fibrosis was assessed via Masson’s trichrome staining (the Fast Green method, Solarbio, China), according to the manufacturer’s instructions. The 5 µm thick lung sections were deparaffinized, stained with Masson’s trichrome solution, and finally observed under a microscope (Nikon, Tokyo, Japan).

### 2.8. Immunohistochemistry

Five-micron-thick lung sections were deparaffinized, treated with H_2_O_2_, and blocked in normal goat serum. The sections were incubated with primary antibodies against CD86 (1:100), p-NFκB (1:100), TGF-β1 (1:200), DRP1 (1:100), MFN1 (1:100), MFN2 (1:100), or PINK1 (1:100), overnight at 4 °C, and then treated with secondary antibodies. A diaminobenzidine detection kit (Solarbio, Beijing, China) was used for color development at room temperature, and the sections were observed under a microscope (Nikon, Y-TV55, Tokyo, Japan). The optical density of each section was quantified using ImageJ software (v1.8.0, Bethesda, MD, USA).

### 2.9. Western Blotting (WB)

Thirty micrograms of protein was subjected to 8–12% SDS-PAGE and transferred to a PVDF membrane. The membranes were blocked in 5% milk for 2 h and incubated with primary antibodies against p-Smad2 Ser476 (1:1000), p-Akt (1:500), and β-actin (1:5000) at 4 °C, overnight. The membranes were incubated with anti-rabbit or anti-mouse IgG antibodies (Abcam, Waltham, MA, USA) at room temperature and then visualized using a commercially available enhanced chemiluminescence kit (Solarbio, China). Band densities were quantified using ImageJ software (v1.8.0, Bethesda, MD, USA). To evaluate the nuclear β-catenin and lamin B1 levels, we separated the nuclear proteins from the rat lungs by using a nuclear kit (BioVision, Milpitas, CA, USA) and then performed WB. To evaluate the expression of mitochondrial LC3, p62, and VDAC1, we separated the mitochondria from the rat lungs by using a mitochondrial kit (C3606, Beyotime, Shanghai, China) and then performed WB.

### 2.10. Enzyme-Linked Immunosorbent Assay (ELISA)

The concentrations of TNF-α, IL-1β, and IL-6 in the lungs of the rats in different treatment groups were measured using ELISA kits (Boster, Wuhan, China), according to the manufacturer’s protocol, after their extraction using a cell lysis buffer.

### 2.11. Real-Time PCR

Total RNA was extracted from the lung tissues by using the TRIpure Reagent (BioTeke, Beijing, China). The RNA concentration of each sample was determined using an ultraviolet spectrophotometer (NANO 200). Total RNA was reverse-transcribed using BeyoRT II M-MLV Reverse Transcriptase, according to the manufacturer’s protocol. The primer information is shown in Table 1. A real-time PCR was performed using an SYBR Green PCR kit (Solarbio, Beijing, China), and the 2^−ΔΔCt^ method was used for the fluorescence quantitative PCR analysis.

### 2.12. Transmission Electron Microscopy

The mitochondrial ultrastructure in the lungs of the rats in different treatment groups was observed using transmission electron microscopy. A 1 mm^3^ sample of the lung tissue was placed in electron microscope fixative for 24 h, at 4 °C. The samples were subsequently fixed in 1% osmium tetroxide for 2 h, dehydrated, embedded in araldite, and polymerized at 37 °C for 8 h, 45 °C for 12 h, and 60 °C for 24 h. Ultrathin slices (70 nm) were stained with uranyl acetate and lead citrate. Finally, a transmission electron microscope (JEOL, Tokyo, Japan) was used at 80 kV for imaging.

### 2.13. Estimation of ATP Levels

Mitochondria were extracted from the rat lungs using a mitochondrial kit (C3606; Beyotime, China). Briefly, pre-cooled mitochondrial separation reagent was added to the lung tissue and homogenized in an ice bath. It was then centrifuged at 1000× *g* at 4 °C for 5 min to remove nuclei and debris. The supernatant was centrifuged at 11,000× *g* at 4 °C for 10 min. The deposition is the separated mitochondria.

Then, the ATP content was subsequently measured using an ATP assay kit (Nanjing Jiancheng Bioengineering Institute, Nanjing, China). Briefly, prepare the standard curve, according to the instructions. Add 100 μL of ATP detection working solution into the detection well of the 96-well plate at room temperature for 5 min to reduce the background signal. Add 20 μL of mitochondrial lysate to each detection well, and measure the RLU (Relative Light Unit) value using a Luminometer. Calculate the concentration of the ATP in the lung mitochondria using the standard curve. Convert the ATP content into μM/g protein, based on the protein concentration.

### 2.14. Analysis of the BMD and BMDL of PS-MP Exposure in Rats

The BMD and BMDL were calculated using Benchmark Dose Tools Online (https://bmdsonline.epa.gov/ (accessed on 3 June 2025)), Environmental Protection Agency, Washington, DC, USA). The doses of PS-MPs were modeled as the predictor variables of the endpoints assessed, and the lung organ coefficient and proinflammatory cytokine contents in the lungs were used as effective biomarkers. The exponential, Hill, linear, polynomial, and power models were used to test the variables. The confidence level was 95%, and the default (benchmark response, BMR) was 5% (continuous data). When the *p* value of the goodness of fit was >0.05, the model was the most fitted and, then, the BMD and BMDL were calculated automatically.

### 2.15. Statistical Analysis

The data are presented as the mean ± SEM. The differences between any two groups were analyzed using a one-way analysis of variance, followed by Fisher’s least significant difference multiple comparison test with homogeneity of variance, or Dunnett’s T3 test with heterogeneity of variance. Statistical significance was set at *p* < 0.05. All the analyses were performed using SPSS 17.0.

## 3. Results

### 3.1. PS-MP Distribution in the Lung Tissues of the Rats

**Figure 2:**
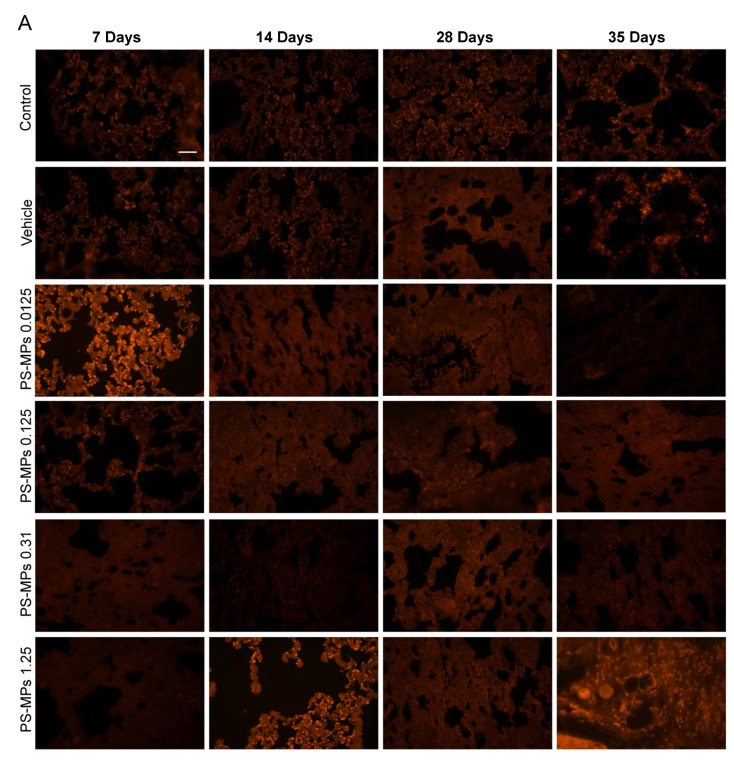

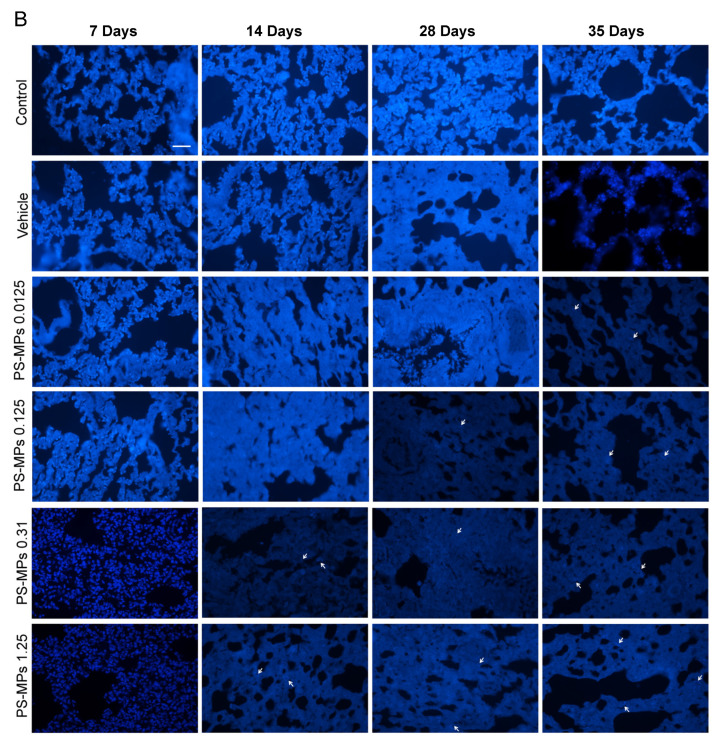

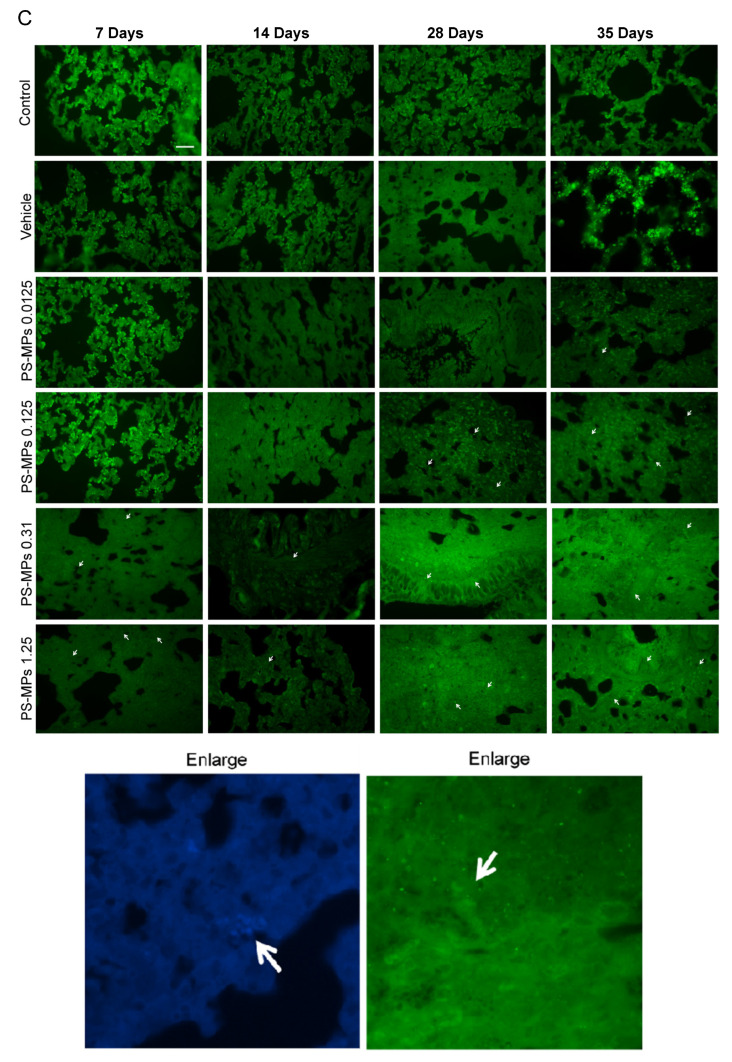
Distribution of PS-MPs in the lungs of rats in different treatment groups: (**A**) no 500 nm PS-MPs were observed; (**B**) 1 and 2 µm PS-MPs were observed; and (**C**) 3 and 4 µm PS-MPs were observed. White arrows indicated the PS-MPs. *n* = 4; bar = 100 μm.

After 7, 14, 28, and 35 days of exposure, we observed the distribution of PS-MPs at three excitation wavelengths (red fluorescence, 548 nm; blue fluorescence, 383 nm; green fluorescence, 484 nm). No PS-MPs were detected in the lung tissues of the rats in the control and vehicle groups; in contrast, many 1 to 4 µm PS-MPs were detected in the lungs of the rats in the PS-MP exposure groups (Figure 2B,C); no 500 nm PS-MPs were detected in the lungs (Figure 2A). However, the fluorescence microscope we used has limited magnification capability, and, in subsequent experiments, we should use a confocal laser microscope to reconfirm this result. The number of PS-MPs in the rat lungs was greater in the 1.25 mg PS-MP exposure group than in the low-dose PS-MP exposure group, and the number of 3 µm and 4 µm PS-MPs was greater than the number of 1 µm and 2 µm PS-MPs.

### 3.2. Intratracheal Administration of PS-MPs Affect the Body and Lung Weights of Rats

**Figure 3:**
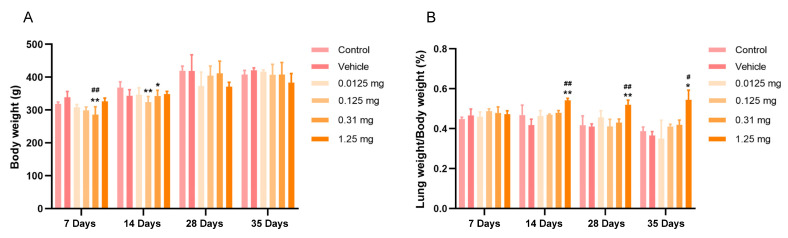
Body weights (**A**) and lung organ coefficients (**B**) of the rats in different treatment groups; *n* = 4. All the results are expressed as the mean ± SD; * *p* < 0.05, ** *p* < 0.01 vs. the control group; # *p* < 0.05, ## *p* < 0.01 vs. the vehicle group.

The body weight of the rats in different treatment groups increased with an increase in the feeding time. When the body weights of the rats were compared, slight decreases were observed in the 0.31 mg PS-MP exposure group after 7 and 14 days of exposure to PS-MPs (Figure 3A). No significant difference in the body weights was observed among the control, vehicle, and PS-MP groups after 28 and 35 days of exposure to PS-MPs (Figure 3A). The lung organ coefficient (the ratio of the lung weight to the body weight) was significantly greater in the rats exposed to PS-MPs than in the rats in the control and vehicle groups (Figure 3B). In particular, after 14 days of PS-MP exposure, the lung organ coefficients significantly increased in the 1.25 mg PS-MP exposure group (*p* < 0.05 and *p* < 0.1). These results suggest that the PS-MPs did not markedly change the weight of the rats, but did significantly affect the lung weight.

### 3.3. Intratracheal Administration of PS-MPs Induces Structural Damage in the Lungs of Rats

**Figure 4:**
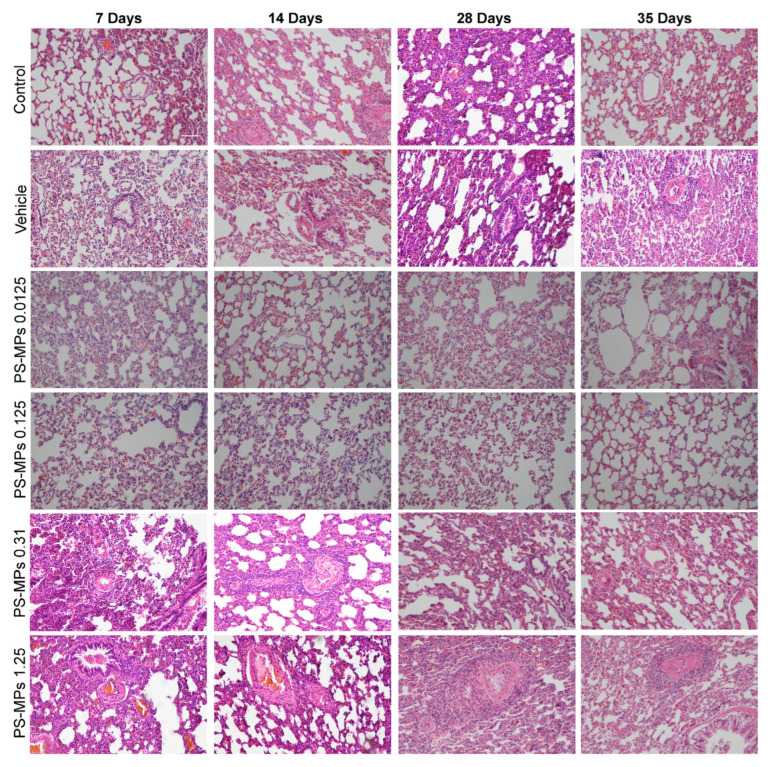
HE staining revealed that the intratracheal administration of the PS-MPs induced damage to the lung structure; *n* = 4; bar = 100 μm.

The lung structure in the different treatment groups was evaluated using HE staining. The alveolar structure was intact in the control, vehicle, 0.0125 mg PS-MP, and 0.125 mg PS-MP groups, the alveolar wall was thin and clear, and the alveolar cavity did not collapse (Figure 4). However, the alveolar structure was irregular in the 0.31 mg and 1.25 mg PS-MP groups; the alveolar wall was thickened, the alveolar cavity collapsed, and there was a large amount of inflammatory cell infiltration (Figure 4). In particular, after 28 and 35 days of exposure to PS-MPs, larger areas of fibrous scars and more severe inflammatory cell infiltration were observed in the lungs of the rats in the 1.25 mg PS-MP group than in those of the rats in the low PS-MP group, indicating that as the PS-MP exposure duration and dose increased, the structural damage to the lungs gradually worsened (Figure 4).

### 3.4. Intratracheal Administration of PS-MPs Activates NFκB-Mediated Pulmonary Inflammation in Rats

**Figure 5:**
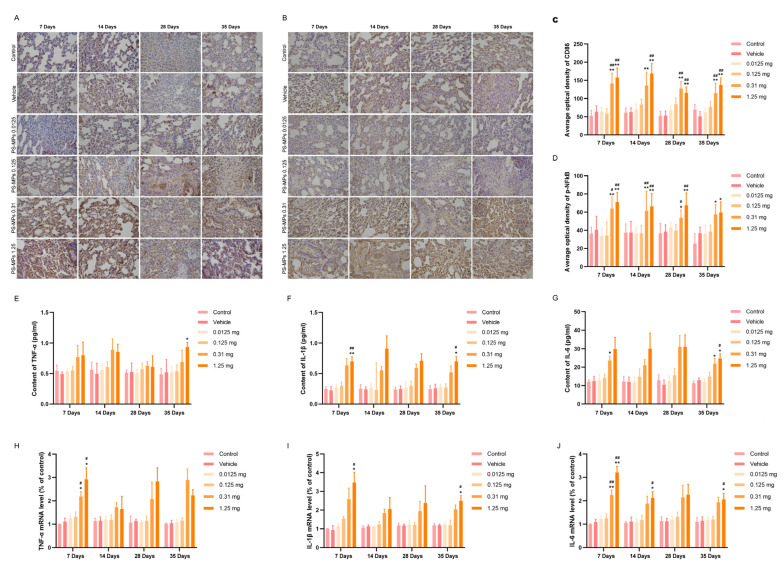
Intratracheal administration of PS-MPs induced pulmonary inflammation in rats. The expression levels of CD86 (**A**,**C**) and p-NFκB (**B**,**D**) increased in the lungs of the rats in the PS-MP exposure groups. The levels of TNF-α (**E**), IL-1β (**F**), and IL-6 (**G**) and the mRNA levels of TNF-α (**H**), IL-1β (**I**), and IL-6 (**J**) increased in the lungs of the rats in the PS-MP exposure groups. All the results are expressed as the mean ± SD; *n* = 4 or 3 (RT PCR); * *p* < 0.05, ** *p* < 0.01 vs. the control group; # *p* < 0.05, ## *p* < 0.01 vs. the vehicle group; bar = 100 μm.

CD86 is a biomarker for M1 macrophages, which secrete proinflammatory cytokines that bind to their receptors and then activate IκBα and phosphorylated IκBα. Nuclear factor κB (NFκB) can be released from the NFκB/phospho inhibitor of κBα (IκBα) complexes, leading to its nuclear translocation; this further promotes the release of proinflammatory cytokines, which can promote the acquisition of the fibroblast phenotype by epithelial cells or endothelial cells [25]. Phosphorylated NF-κB promotes the nuclear translocation of p50/p65 and p65 NF-κB; therefore, the phosphorylation of NF-κB can be used as an indicator of nuclear transcription levels [26,27,28]. The results revealed that the expression levels of CD86 and p-NFκB were significantly greater in the lungs of rats in the 0.31 mg/kg and 1.25 mg/kg PS-MP group than in those of the control and vehicle groups, with the highest expression occurring at 7 and 14 days of exposure (Figure 5A–D). However, the difference in the expression of CD86 and p-NFκB between the 0.31 mg and 1.25 mg PS-MP groups was minimal. Compared with those in the control and vehicle groups, the protein and mRNA levels of TNF-α, IL-1β, and IL-6 in the low PS-MP (0.0125 mg and 0.125 mg) exposure groups showed little change (Figure 5E–J). In the high PS-MP (0.31 mg and 1.25 mg) exposure groups, the protein and mRNA levels of TNF-α, IL-1β, and IL-6 clearly increased, especially on the 7th day and 35th day after exposure to PS-MPs (Figure 5E–J). The peak proinflammatory cytokine levels were detected on the 7th day after exposure to PS-MPs, which indicated that proinflammatory cytokines may be sensitive indicators of lung injury.

### 3.5. Intratracheal Administration of PS-MPs Activates TGF-β1/Smad Signaling to Promote Pulmonary Fibrosis in Rats

**Figure 6:**
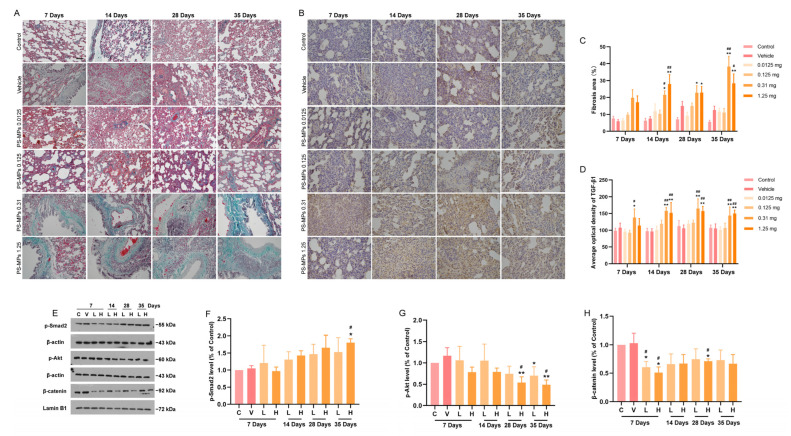
Intratracheal administration of PS-MPs induced pulmonary fibrosis in rats. Masson’s trichrome staining revealed green collagen deposition in the rat lungs (**A**,**C**). The expression levels of TGF-β1 (**B**,**D**) and p-Smad2 (**E**,**F**) increased, and the expression levels of p-Akt (**E**,**G**) and nuclear β-catenin (**E**,**H**) decreased in the lungs of the rats in the PS-MP exposure groups. Abbreviations for WB analysis are C: control, V: vehicle, L: 0.31 mg PS-MPs, H: 1.25 mg PS-MPs. All the results are expressed as the mean ± SD; *n* = 4. * *p* < 0.05, ** *p* < 0.01 vs. the control group; # *p* < 0.05, ## *p* < 0.01 vs. the vehicle group; bar = 100 μm.

The ECM forms a three-dimensional scaffold on the alveolar wall. Abnormal ECM deposition induces endothelial cell dysfunction and structural damage. Excessive ECM deposition involves different types of collagen, which is an important pathological marker of pulmonary fibrosis and can be stained green with Masson’s trichrome solution. Masson’s trichrome staining of the lungs of the rats in different treatment groups revealed no significant green collagen deposition in the lungs in the control group after 35 days (Figure 6A,C). Compared with the control group, the vehicle group presented a small amount of green collagen deposition in the lungs after 28 days, but the difference was not statistically significant (Figure 6A,C). Green collagen deposition in the rat lungs was obvious and increased significantly with the increasing PS-MP exposure duration in the PS-MP exposure groups (Figure 6A,C). These results indicated that high doses (0.31 mg and 1.25 mg) of PS-MPs induced pulmonary fibrosis, and the degree of pulmonary fibrosis increased with the increasing PS-MP exposure duration.

Transforming growth factor-β1 (TGF-β1) is a key factor that affects fibrosis, immunomodulation, and the subsequent regulation of the epithelial–mesenchymal transition (EMT) [29]. The action of TGF-β1/small mothers against decapentaplegic (SMAD) protein signaling can regulate the PI3K/Akt and Wnt/β-catenin pathways [30], which are involved in the fibrosis process. The results of the WB analysis of the lungs of rats in different groups suggested that the TGF-β1 level in the lungs of rats exposed to high-dose PS-MPs was significantly increased at 7, 14, 28, and 35 days (Figure 6B,D–F). In addition, the p-Smad2 level in the lungs of the rats exposed to 1.25 mg of PS-MPs significantly increased at 35 days, and the p-Akt and nuclear β-catenin levels significantly decreased with prolonged high-dose PS-MP exposure (Figure 6E,G,H). No significant differences in the TGF-β1 and p-Smad2 levels were detected between the 0.31 mg and 1.25 mg PS-MP exposure groups.

### 3.6. Mitochondrial Dynamics and Mitophagy Dysfunction Aggravate Pulmonary Fibrosis Induced by PS-MPs

Mitochondria are the most important cellular organelles for regulating biological energy generation and intracellular calcium imbalance, etc. [31,32,33]. Mdivi-1, a DRP1 inhibitor, can prevent PM_2.5_-induced death in alveolar epithelial cell lines (A549 cells) [34]. MFN1 and MFN2 knockout aggravates bleomycin-induced pulmonary fibrosis in mice [35]. Thus, an imbalance between mitochondrial fission and fusion is one of the causes of pulmonary fibrosis. Fusion causes mitochondria to form a network; fission separates damaged mitochondria from the network, and they are then cleared as a result of mitophagy. Mitophagy is a type of autophagy that automatically eliminates damaged or dysfunctional mitochondria. If damaged intracellular mitochondria cannot be removed immediately, cytochrome c is released from the mitochondria, further inducing apoptosis.

**Figure 7:**
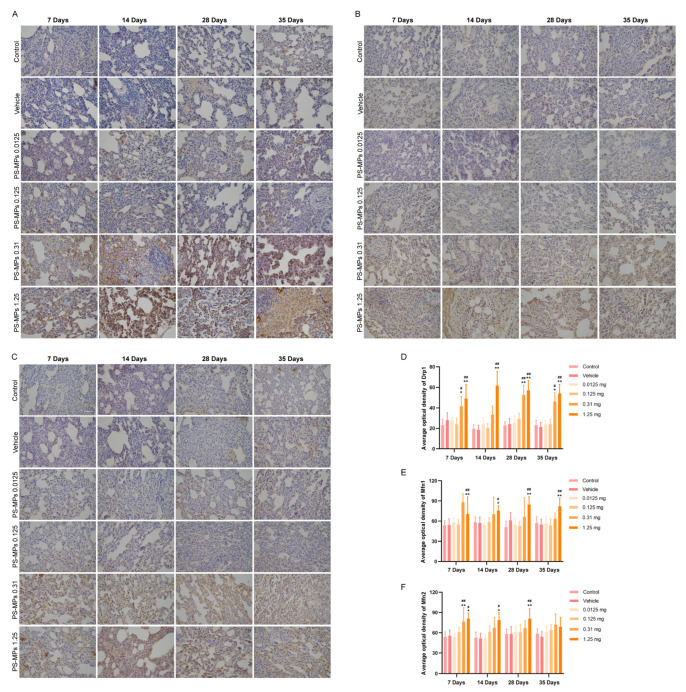
Mitochondrial fission and fusion proteins in the lungs of rats in different treatment groups. Intratracheal administration of PS-MPs increased the expression of Drp1 (**A**,**D**), Mfn1 (**B**,**E**), and Mfn2 (**C**,**F**) in the lungs of rats. All the results are expressed as the mean ± SD; *n* = 4; * *p* < 0.05, ** *p* < 0.01; # *p* < 0.05, ## *p* < 0.01 vs. the vehicle group; bar = 100 μm.

In this study, the expression of the fission protein, Drp1, was greater in the lungs of the rats in the 0.31 mg and 1.25 mg PS-MP exposure groups than in those of the control and vehicle groups on the 7th, 28th, and 35th days after exposure to PS-MPs (Figure 7A,D). The expression of the fusion protein, Mfn1, was greater in the lungs of the rats in the 1.25 mg PS-MP exposure group than in those of the control and vehicle groups on the 7th, 14th, 28th, and 35th days after exposure to PS-MPs (Figure 7B,E). Mfn2 expression was greater in the lungs of the 1.25 mg PS-MP exposure group on the 7th, 14th, and 28th days after exposure to PS-MPs (Figure 7C,F) than in those of the control and vehicle groups.

**Figure 8:**
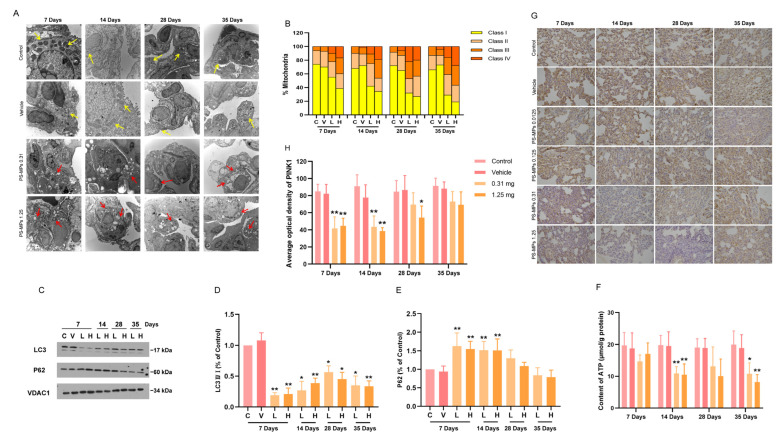
Intratracheal administration of PS-MPs induced changes in pulmonary mitochondrial structure and function. The results of the mitochondrial ultrastructure analysis revealed fewer healthy mitochondria (class I and class II) and more unhealthy mitochondria (class III and class IV) in the lungs of the PS-MP exposure groups (**A**,**B**). Yellow arrows indicated class I and class II mitochondria. Red arrows indicated the PS-MPs class III and class IV mitochondria. The mitochondrial expression of LC3Ⅱ decreased (**C**,**D**), that of P62 increased (**C**,**E**), the ATP content decreased (**F**), and PINK1 expression decreased (**G**,**H**) in the lungs of the PS-MP exposure groups. Abbreviations for WB analysis are C: control, V: vehicle, L: 0.31 mg, H: 1.25 mg. All the results are expressed as the mean ± SD. Quantification of the mitochondrial ultrastructure was performed using at least 100 mitochondria. For the other results: *n* = 4; * *p* < 0.05, ** *p* < 0.01 vs. the control group; bar (TEM) = 1 μm; bar (IHC) = 100 μm.

Vaillant-Beuchot L et al. (2021) divided mitochondrial morphology into four categories (class I: mitochondria with a uniform matrix filled with densely packed, regularly distributed cristae; class II: mitochondria with disrupted cristae and a reduced matrix density; class III: empty mitochondria with disorganized cristae or cristae at the periphery; and class IV: swollen mitochondria with disrupted membranes) [36]. In this study, the ultrastructural characteristics of mitochondria in the rat lungs revealed that the mitochondria were healthy (class I and class II) in the control and vehicle groups (Figure 8A,B). With a prolonged PS-MP exposure duration and an increasing PS-MP exposure dose, the number of healthy mitochondria (class I and class II) decreased, and the number of unhealthy mitochondria (class III and class IV) increased in the rat lungs (Figure 8A,B, Supplementary Figure S1).

The mitochondria were extracted from the lungs to measure the ATP, microtubule-associated protein light chain 3 (LC3), and autophagosome substrate sequestosome-1 (SQSTM1/p62) contents. PINK1/Parkin is the main signaling pathway that mediates mitophagy [37]. The expression of LC3Ⅱ (Figure 8C,D) and PINK1 (Figure 8G,H) and the ATP content (Figure 8F) were significantly reduced in the lungs of the PS-MP exposure groups, but the expression of P62 was significantly increased (Figure 8C,E). As the PS-MP exposure duration increased, the changes in LC3Ⅱ, P62, ATP, and PINK1 expression differed from those observed after exposure to PS-MPs for 7 days, and the expression of LC3Ⅱ slightly increased after exposure to PS-MPs for 28 days (Figure 8D). The P62 level first increased significantly, but then decreased gradually (Figure 8E), and the ATP content gradually decreased (Figure 8F). Compared with the results for the exposure to PS-MPs for 7 days, the expression of PINK1 gradually increased after exposure to PS-MPs for 28 and 35 days (Figure 8H).

### 3.7. Assessment of the BMD and BMDL by Evaluating the Lung Organ Coefficient and Proinflammatory Cytokine Contents in Rats Subjected to PS-MPs

On the basis of the exponential, Hill, linear, polynomial, and power models, the Akaike information coefficient (AIC), *p* value, BMD, and BMDL were calculated, and are presented in Table 2. According to the goodness-of-fit test *p* value (>0.1) and minimal AIC, the best-fit model for the PS-MPs by intratracheal administration for 7 days was the ExponentialM5 model of the lung IL-1β content; the BMD that induced lung injury was 0.141 mg/day, and the BMDL was 0.095 mg/day. The best-fit model for 35 days was the power model of the lung TNF-α content; the BMD that induced lung injury was 0.151 mg/day, and the BMDL was 0.031 mg/day. The toxicological endpoint with the most conservative BMDL value from the animal studies was identified. Therefore, a BMDL value of 0.031 mg was an acceptable daily intratracheal administration dose for rats.

## 4. Discussion

Atmospheric MPs can be directly and continuously inhaled into lung tissue and then accumulate in the lungs or enter the circulatory system through alveolar capillary barriers, depending on the MP particle size [7,9,38]. MPs with particle sizes greater than 30 μm are less likely to be inhaled [39]. MPs ≤10 µm can reach the human respiratory system and accumulate in the upper airways or can enter the intrathoracic cavity [40,41,42]. In this study, after exposure for 7, 14, 28, or 35 days, more 1 to 4 µm PS-MPs were observed in the lungs of the rats in the PS-MP exposure groups. Moreover, 500 nm PS-MPs were not observed in this experiment, due to the small particle size. In subsequent experiments, we will try other methods for observation. The literature has reported that the intracellular uptake of PS-MPs has been observed in many cells [43,44,45,46]. In regard to immunohistochemistry, we washed the lung tissue sections with a large amount of PBS buffer and distilled water, which was able to remove the PS-MPs on the cell surface. Therefore, we believe that the PS-MPs were in the cytoplasm. However, more convincing experimental methods (such as z-stacks) will be used to verify the distribution of PS-MPs in the cytoplasm or on the cell surface in our future research. However, the intratracheal aerosolization route is better than instillation. But this method still has limitations, for example, the intratracheal administration volume is restricted, to only 50–70 µL per use for rats; successful intratracheal administration is dependent on the breathing rate of the animal; therefore, the distribution of MPs in the lung is not homogeneous; MPs with a particle size >5 µm are not suitable for intratracheal administration.

Pulmonary fibrosis is characterized by the proliferation of fibroblasts and the accumulation of excess ECM, accompanied by inflammation and damage to the tissue structure. External stimuli can activate macrophages in the lungs, further inducing epithelial cells or endothelial cells to release TGF-β1 and promote fibrosis [25,47]. During lung damage, activated M1 macrophages secrete proinflammatory cytokines, chemokines, and adhesion molecules. These substances can promote lung epithelial cell differentiation and the inflammatory response and activate the NF-κB transcription factor located outside the nucleus to enter the nucleus to promote inflammation and immune responses in the cells [25]. Repeated alveolar epithelial cell injury leads to an overload of secreted cytokines, inducing alveolar epithelial type II (AT-II) transformation into a mesenchymal cell phenotype and differentiation into fibroblasts through EMT [48,49]. Fibroblasts are further converted into myofibroblasts, which secrete ECM and lead to alveolar collapse and lung dysfunction [50]. TGF-β1 binds to the TGF-β1 type II receptor and recruits the TGF-β1 type I receptor, leading to a transcriptional response from Smad-responsive target genes [51].

The inhalation of MPs can cause lung tissue lesions and infections [9,52,53,54,55]. In this study, pulmonary fibrosis was very severe, and the rat lungs presented obvious green collagen deposition, an irregular alveolar structure, a thickened alveolar wall, a collapsed alveolar cavity, and extensive inflammatory cell infiltration. In particular, after 28 and 35 days of exposure to PS-MPs, large areas of fibrous scars and more severe inflammatory cell infiltration were detected in the lungs of the rats in the high PS-MP exposure group. The proinflammatory cytokine levels, the expression of CD86 (a biomarker of M1 macrophages), and NFκB and TGF-β1/Smad signaling were significantly increased in the rats exposed to PS-MPs, but the p-Akt and nuclear β-catenin levels in the lungs of the rats were decreased. These results indicated that the intratracheal administration of PS-MPs induced pulmonary fibrosis in the rats, which may be related to the release of proinflammatory cytokines by PS-MP-activated macrophages and the activation of TGF-β1 and its downstream signals in the lungs.

Canonical Wnt signaling depends on β-catenin activity. Stabilized β-catenin accumulates in the cytosol; when β-catenin is activated, it is translocated into the nucleus, where it stimulates the transcription of target genes, such as Snail and PAI-1, which participate in fibrogenesis [53]. However, in this study, the nuclear β-catenin level decreased, which was unexpected. Other signals may also regulate β-catenin during the induction of pulmonary fibrosis by PS-MPs, but this topic needs to be studied further.

Mitochondria are the most important cellular organelles involved in the regulation of biological energy generation and intracellular calcium imbalance, etc. [31,32,33]. Mitochondrial damage is a pathological change in regard to pulmonary fibrosis. Mitochondrial electron transport chain dysfunction, inefficient mitophagy, and inhibited biogenesis and ATP synthesis have been observed in pulmonary fibrosis [56,57,58]. In this study, PS-MP exposure significantly decreased the ATP content in the lungs. PINK1 is a mitochondria-targeting protein with kinase activity. When mitochondria are damaged, PINK1 is recruited, it accumulates on the mitochondrial outer membrane, and activates the E3 ubiquitin ligase activity of Parkin, which ubiquitinates and triggers the expression of select autophagy-related mitochondrial proteins. PINK1/Parkin-mediated mitophagy can rapidly clear damaged mitochondria, prevent the apoptosis induced by cytochrome C release, regulate mitochondrial dynamics, and regulate mitochondrial fission, fusion, degradation, and transportation [59]. In this study, PS-MP exposure significantly inhibited PINK1-mediated mitophagy and blocked homeostasis and autophagic flux in the lungs.

Under physiological conditions, mitochondrial fission and fusion occur in equilibrium. The knockdown of the mitochondrial fusion protein Mfn1/2 in lung AT-II cells increased the morbidity and mortality of pulmonary fibrosis in mice, because Mfn1/2 knockdown impaired lipid metabolism and synthesis and significantly decreased the number and area of the mitochondria [35]. In chronic or acute pulmonary disease, the levels of mitochondrial dynamics-related proteins were reported to significantly change [60,61]. In this study, with a prolonged exposure duration and increased exposure dose, the expression of the fission protein, Drp1, and the fusion protein, Mfn1/2, significantly increased after exposure to PS-MPs, indicating that the mitochondrial dynamics gradually became chaotic. Thus, destabilizing mitochondrial dynamics and mitophagy may aggravate PS-MP-induced pulmonary fibrosis.

The BMD method has been used to evaluate the health risk of environmental pollutants and is more sophisticated and robust than the no observed adverse effect level (NOAEL) method [62]. The US National Academy Committee suggests that the BMD should be the preferred approach for chemical hazard exposure assessments [63]. Calculating the dose-response relationships using mathematical models is very important for evaluating the toxicity of environmental hazards [64]. Taft et al. (2012) used the BMD method to investigate the toxicity of inhaled Bacillus anthracis in nonhuman primates [65]. Blum et al. (2023) used the BMD method to derive occupational exposure limits for inhaled N-nitrosamines [66]. Rogers et al. (1993) used the BMD method to test the developmental toxicity of inhaled methanol in CD-1 mice [67]. In this study, when the lung organ coefficient and proinflammatory cytokine contents in the lungs were used as effective biomarkers, there was a significant dose-response relationship between lung injury and the PS-MP exposure dose, especially 35 days after the PS-MP exposure. A BMD of 0.151 mg/day and a BMDL of 0.031 mg/day were used to measure the subchronic effects of the intratracheal administration of PS-MPs in rats. To our knowledge, this study is the first to use BMDs to evaluate the threshold at which PS-MPs induce lung injury in rats.

## 5. Conclusions

In summary, rats were given mixed MPs with four particle sizes by intratracheal administration for 7, 14, 28, and 35 consecutive days. This treatment gradually induced severe pulmonary fibrosis and inflammation and damaged the structure of the lung mitochondria. Increasing TGF-β1 signaling and destabilizing mitochondrial dynamics and mitophagy may aggravate pulmonary fibrosis induced by PS-MPs. In accordance with the best-fit model, the BMD and BMDL of the intratracheal administration of PS-MPs were calculated for the first time.

## Figures and Tables

**Table 1:** Primer information for PCR analyses.

Genes	Primer Sequences
TNF-α	F: TGTTCATCCGTTCTCTACCCA
	R: CACTACTTCAGCGTCTCGT
IL-1β	F: CTATGGCAACTGTCCCTGAA
	R: GGCTTGGAAGCAATCCTTAATC
IL-6	F: GAAGTTAGAGTCACAGAAGGAGTG
	R: GTTTGCCGAGTAGACCTCATAG
β-actin	F: TGCTATGTTGCCCTAGACTTCG
	R: GTTGGCATAGAGGTCTTTACGG

**Table 2:** BMD and BMDL estimation of lung organ coefficient and proinflammatory cytokine contents.

Time Point		Model of Fit	Goodness of Fit *p* Value	AIC	BMD	BMDL
	Parameters					
7 Days						
Lung organ coefficient (%)		NDR	NDR	NDR	NDR	NDR
TNF-α (pg/mL)		NDR	NDR	NDR	NDR	NDR
IL-1β (pg/mL)		ExponentialM5	0.545	−45.136	0.141	0.095
IL-6 (pg/mL)		NT	NT	NT	NT	NT
14 Days						
Lung organ coefficient (%)		NT	NT	NT	NT	NT
TNF-α (pg/mL)		NDR	NDR	NDR	NDR	NDR
IL-1β (pg/mL)		NDR	NDR	NDR	NDR	NDR
IL-6 (pg/mL)		NDR	NDR	NDR	NDR	NDR
28 Days						
Lung organ coefficient (%)		NT	NT	NT	NT	NT
TNF-α (pg/mL)		NDR	NDR	NDR	NDR	NDR
IL-1β (pg/mL)		NDR	NDR	NDR	NDR	NDR
IL-6 (pg/mL)		NDR	NDR	NDR	NDR	NDR
35 Days						
Lung organ coefficient (%)		NT	NT	NT	NT	NT
TNF-α (pg/mL)		Power	0.594	−24.876	0.151	0.031
IL-1β (pg/mL)		ExponentialM5	0.490	−43.294	0.205	0.115
IL-6 (pg/mL)		Polynomial 2°	0.538	91.815	0.05	0.037

Notes: NDR means no dose response detected; NT means that none of the models fit.

## Data Availability

The data are available in a publicly accessible repository, https://pan.baidu.com/s/1P-j1Uxe2sa483CrVzP9HcQ?pwd=6a56 (accessed on 3 June 2025).

## Supplementary Materials

The following supporting information can be downloaded at: https://www.mdpi.com/article/10.3390/toxics13060487/s1, Figure S1: Enlarged images of mitochondria in the lungs of the PS-MP exposure groups.

## Abbreviations

The following abbreviations are used in this manuscript:

Akt	protein kinase B
AT-II cell	alveolar epithelial type II cell
BMD	benchmark dose
BMDL	benchmark dose lower confidence limit
Drp1	dynamic associated protein 1
EMT	epithelial mesenchymal transition
ECM	extracellular matrix
IκBα	Phospho inhibitor of κBα
IL-1β	interleukin-1β
IL-6	interleukin-6
LC3	microtubule-associated protein light chain 3
Mfn1/2	mitochondrial fusion 1/2
MPs	microplastics
NFκB	nuclear factor κB
NOAEL	no observed adverse effect level
NPs	nanoplastics
PI3K	phosphatidylinositol-4,5-bisphosphate 3-kinase
PINK1	PTEN-induced putative kinase 1
PS-MPs	polystyrene microplastics
SMAD	small mothers against decapentaplegic proteins
SQSTM1/p62	sequestosome-1
TGF-β1	transforming growth factor-β1
TNF-α	tumor necrosis factor α
