# Supplementary figures and images for: Intratracheal Administration of Polystyrene Micro(nano)plastics with a Mixed Particle Size Promote Pulmonary Fibrosis in Rats by Activating TGF-β1 Signaling and Destabilizing Mitochondrial Dynamics and Mitophagy in a Dose- and Time-Dependent Manner
Source: Toxics. 2025 Jun 9;13(6):487. doi: 10.3390/toxics13060487 (PMC12197594; doi:10.3390/toxics13060487)

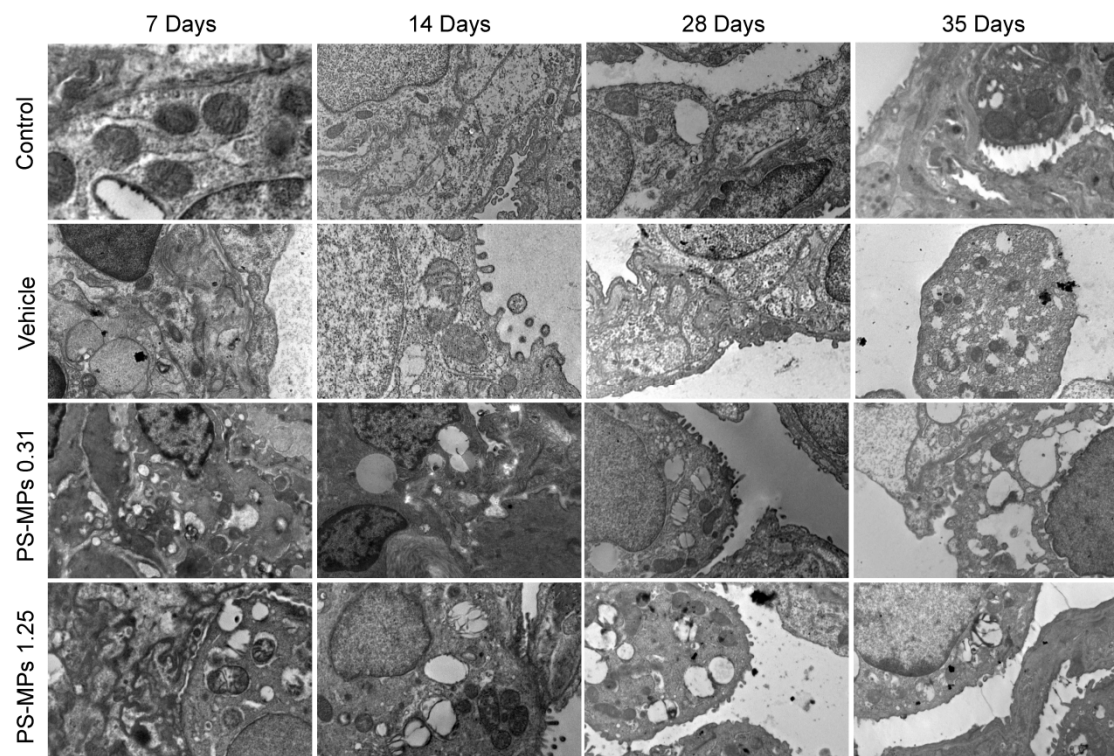

**Figure S1.** The enlarged images of mitochondria in the lungs of the PS-MP exposure groups.

Supplement: Supplementary file 1 [file toxics-13-00487-s001.zip › toxics-3589733-supplementary.pdf]
